# Survival time prediction by integrating cox proportional hazards network and distribution function network

**DOI:** 10.1186/s12859-021-04103-w

**Published:** 2021-04-15

**Authors:** Eu-Tteum Baek, Hyung Jeong Yang, Soo Hyung Kim, Guee Sang Lee, In-Jae Oh, Sae-Ryung Kang, Jung-Joon Min

**Affiliations:** 1 Smart Mobility Materials and Components R&D Group, Seonam Division, Korea Institute of Industrial Technology, Gwangju, South Korea; 2 Department of Artificial Intelligence Convergence, Chonnam National University, Gwangju, South Korea; 3 Department of Internal Medicine, Chonnam National University Medical School, Jeonnam, South Korea; 4 Department of Nuclear Medicine, Chonnam National University Medical School, Jeonnam, South Korea; 5 The Institute for Molecular Imaging and Theranostics, Chonnam National University Medical School, Jeonnam, South Korea

**Keywords:** Prognosis, Survival time prediction, Deep learning, Distribution function network, Cox proportional hazards network

## Abstract

**Background:**

The Cox proportional hazards model is commonly used to predict hazard ratio, which is the risk or probability of occurrence of an event of interest. However, the Cox proportional hazard model cannot directly generate an individual survival time. To do this, the survival analysis in the Cox model converts the hazard ratio to survival times through distributions such as the exponential, Weibull, Gompertz or log-normal distributions. In other words, to generate the survival time, the Cox model has to select a specific distribution over time.

**Results:**

This study presents a method to predict the survival time by integrating hazard network and a distribution function network. The Cox proportional hazards network is adapted in DeepSurv for the prediction of the hazard ratio and a distribution function network applied to generate the survival time. To evaluate the performance of the proposed method, a new evaluation metric that calculates the intersection over union between the predicted curve and ground truth was proposed. To further understand significant prognostic factors, we use the 1D gradient-weighted class activation mapping method to highlight the network activations as a heat map visualization over an input data. The performance of the proposed method was experimentally verified and the results compared to other existing methods.

**Conclusions:**

Our results confirmed that the combination of the two networks, Cox proportional hazards network and distribution function network, can effectively generate accurate survival time.

## Background

There have been remarkable technological advances in multiple field of deep learning that are influencing various sectors such as automatic speech recognition, image recognition, customer relationship management, financial, and also the medical field. The advances in deep learning technologies have allowed for improvement in the existing medical technologies and their capabilities. However, aspects such as the accurate prediction of survival times (time to event) in patients remains a challenge [1].

Survival analysis is a part of statistics for analyzing the likely duration until the occurrence of an event of interest. In the medical field, these events may be death, cardiac arrest, the occurrence of a disease, etc. Generally, the survival times are quantified in days, weeks, months, and years. For example, if the event of interest is death, the survival time could be the years until an individual’s death. In addition, survival analysis consists of two fundamental functions: the survival function and the hazard function [2]. The survival function, denoted by S(t) = P (T > t), indicates the probability that an individual has “survived” beyond time t. The hazard function is a measure of risk at time t. A greater hazard ratio signifies a greater risk of death. In previous studies, the hazard ratio was predicted by learning the relationship between covariates and the coefficients of the model using a hazard function.

The analysis of chances of survival is usually a difficult process due to censoring. Censoring is when the study ends, or participants drop out of the study before occurrence of the event, leaving incomplete information about the survival time. Right censoring is the most common type of censoring, which occurs when the participant fails to record any events of interest during the study period, and when the last observed follow-up time earlier the event occurrence time. This problem results in lack of information such that ordinary regression models for survival cannot be applied [3, 4].

Cox proportional hazard regression model is the commonly used hazard model in the medical field since it deals with censoring [5]. The Cox model investigates the association between covariates and the survival time of patients to predict hazard ratio while handling the censoring of observations. In addition, Cox model is a semi-parametric model, therefore it makes no assumptions about the form of the baseline hazard function, however, it cannot directly predict the survival time since it is a type of hazard model. A specific distribution is therefore selected to generate survival time [6], which is problematic since the user must pre-select the distribution manually.

A survival time prediction deep neural network by integration of the Cox hazard ratio network [7] and a distribution function network was proposed. The following contributions were made: First, a distribution function network with a new loss function, which measures the discrepancy between the algorithm's prediction and the desired output was made. Second, the Cox hazard ratio network was integrated with the distribution function network. Finally, the proposed model was made to learn the definite distribution on several datasets.

The survival analysis is commonly used to predict prognosis in patients. After the introduction of the Kaplan–Meier survival estimator [8] and the Cox hazard model [5], numerous studies have been conducted based on these methods. Recently, a significant improvement in performance was observed as a result of deep learning concepts introduced in the medical field. The survival methods were classified as shown in Table 1, and the relevant related works and state-of-the-art studies reviewed. Also, the limitations that cover the problem domain were discussed.

**Table 1:** Taxonomy for survival estimation

Prognosis category	Hazard estimation		Survival estimation		
Machine learning category	Hazard estimation (regression)		Survival time estimation (regression)	Survival status (classification)	
Method	Non-deep learning	Deep learning	Non-deep learning	Non-deep learning	Deep learning
Algorithms	Penalized Cox model [9] Cox-Boost algorithm [10] Time-Dependent Cox model [11] Lasso-Cox model [12]	Deep exponential families [13] DeepSurv [1] DeepHit [14]	Exponential distribution [15, 16] Weibull distribution [17] Gompertz distribution [18] Log-logistic distribution [19]	Support vector machine [20] Decision tree [21]	Lung tumor with deep learning [22] Brain tumor with deep learning [23]

Cox hazard model estimates the hazard ratio for an individual and measures the effect of survival on patients’ covariates in the model. Various modified Cox models have been suggested, for example, the penalized Cox model [9], the Cox-Boost algorithm [10], the Time-Dependent Cox model [11], and Lasso-Cox model [12]. Hazard-predicting methods have received substantial attention in deep learning method. The recently developed models include deep exponential families [13], DeepSurv [1], and DeepHit [14]. DeepSurv attempts to implement modern deep learning techniques to the Cox proportional hazards loss function. It predicts a patient’s risk of death through multi-layered perceptron, hence outperforms other conventional methods. However, to generate an individual’s survival time, a specific distribution should be selected. Survival estimation is utilized to measure the fraction of patients living. Kaplan–Meier estimator is one of the most popular survival estimation methods. It predicts the survival distribution function from censored data, but does not incorporate the patient’s covariates, hence cannot be applied for predicting survival time of an individual. The previous approaches have solved this problem in two main steps: (1) extracted hazard ratio and specific distribution are used to generate the survival time. (2) features are directly used to predict the survival time of an individual.

The approaches in the first step translate hazard ratio to survival time using specific distribution. The commonly applied simulation approaches consider exponential distribution for convenience [15, 16]. In addition, various distributions are used, e.g. normal, Weibull, Gompertz, and log-logistic distributions [16–19]. However, the above-mentioned methods are cumbersome and inaccurate since users must manually select the distributions.

The second approaches apply machine learning methods to estimate the survival time. Survival tree is a method that is specifically customized to handle censored data [24, 25]. The tree recursively partitions based on a particular splitting criterion and similarities to each other. Support vector machine [26, 27] was used for prognosis through reconstruction of the survival estimation as a classification, dividing the time axis by a pre-defined interval or class. Application of SVM were regarded as regression problems in previous studies. Deep learning methods are utilized in the extraction of meaningful features from medical data and in the prediction of the prognosis [20, 22, 28]. They present better performances compared to traditional methods.

We address the method of directly predicting survival time while dealing with censoring data. Thereby, we overcome the disadvantage of not directly estimating survival time from censoring data.

## Results

The performance of the proposed method was evaluated through comparison with the existing survival generation functions in analyzing four real-world clinical datasets. In addition, to assess the proposed distribution function network, the results were visually evaluated through generation of graphs for each dataset.

### Data sets

*Lung1 data set*: The Lung1 dataset contains clinical data and computed tomography (CT) from 422 patients with non-small cell lung cancer (NSCLC) who received radiotherapy [29]. However, only clinical data without CT was used in this study. The clinical data contains 7 variables namely: T-stage, N-stage, M-stage, overall-stage, histology, gender, and age. To use categorical variables, we used label encoder to convert the variables to numerical numbers.

*METABRIC*: This collection contains gene expression and long-term clinical follow-up data of 2059 primary breast tumors [30]. Of the total 2059 patients, 361 patients without clinical data were removed and the experiment was conducted using the data of 1698 patients. The focus was on 15 clinical features namely; the number of positive lymph node, Nottingham prognostic index (NPI), cellularity, chemotherapy, estrogen receptor (ER) status measured by immunohistochemistry, human epidermal growth factor receptor 2 (HER2) status, HER2 status measured by single nucleotide polymorphism 6 (SNP6), ER status, integrative cluster, inferred menopausal state, histologic grade, hormone therapy, histological subtype, location of the tumor, and the 3-Gene classifier subtype.

*Heart disease data set*: Heart disease database contains 76 attributes; however, 13 subset attributes were used for experiments similar to [31–33]. Out of the 13 attributes, 4 were continuous and 8 discrete attributes. The attributes consisted of age, sex, chest pain type, resting blood pressure, serum cholesterol (mg/dL), fasting blood sugar > 120 mg/dL, resting electro-cardio graphic results, maximum heart ratio achieved, exercise induced angina, old peak (ST depression induced by exercise relative to rest), the slope of the peak exercise ST segment, and the number of major vessels (0–3) colored by fluoroscopy. The individuals were categorized into five levels of heart disease. Three of the discrete attributes had two levels, three of the discrete attributes had three levels and two of the discrete attributes had four levels. To generate more accurate estimations, each attribute was rescaled to between 0 and 1.

*PBC data set*: Primary biliary cirrhosis (PBC) data set consisted of clinical data of primary biliary cirrhosis, a rare autoimmune liver disease [34]. 424 patients’ data was obtained from a Mayo Clinic trial in PBC of liver conducted between 1974 and 1984. The focus was on age, sex, edema, bilirubin concentration, albumin concentration, prothrombin time, and disease stage. The variables were used after converting categorical values to numerical values.

### Performance metric

**Fig. 1:**
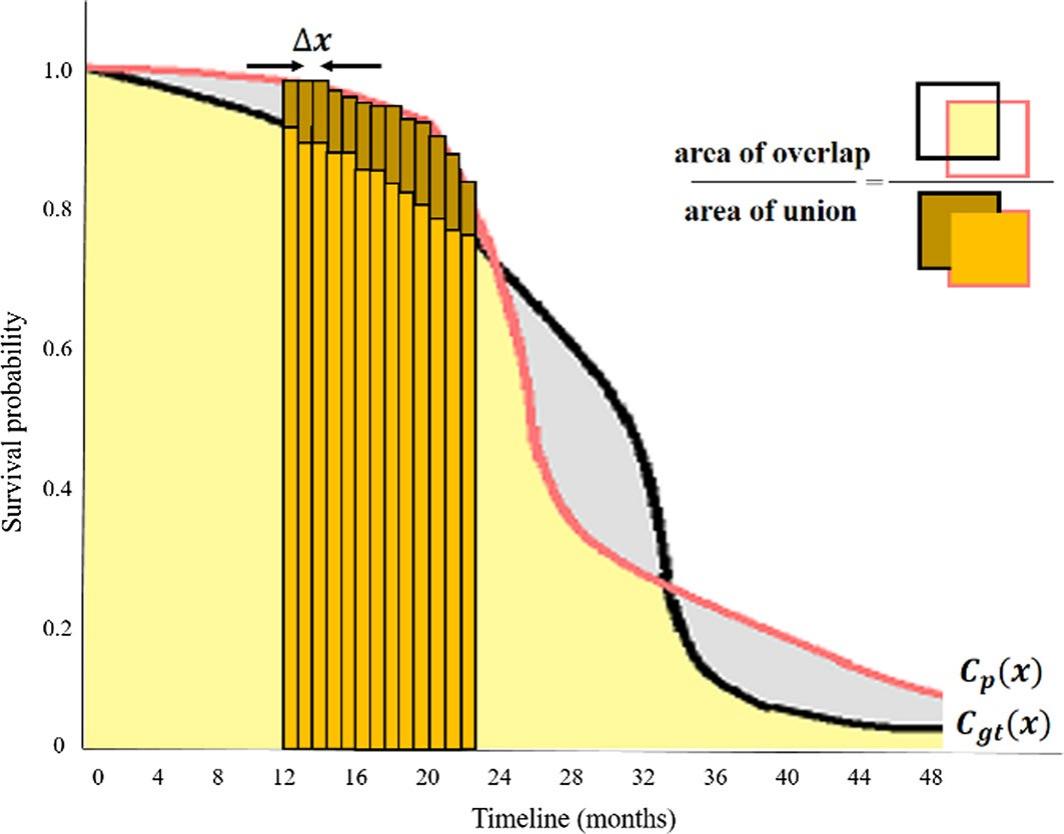
The example of IoU between curves. The y-axis indicates the survival probability, while the x-axis represents the timeline. To evaluate the area under curve, line integral was calculated

The proposed method was evaluated quantitatively and visually on several datasets, and comparison performed with the general functions of other conventional survival time. Since the existing evaluation methods do not allow for the censoring data, it was difficult to evaluate the proposed method through conventional evaluation methods. A new evaluation method was suggested for intersection over union (IoU) between two Kaplan–Meier curves. First, two Kaplan–Meier curves of ground truth and estimation result are plotted. The Kaplan–Meier curve is preferred because it can take into account right censoring. After plotting the two curves, IoU is calculated to estimate the overlap between the two curves. The IoU is defined as follows:

1 $$IoU = \frac{{area\left( {C_{p} \cap C_{gt} } \right)}}{{area\left( {C_{p} \cup C_{gt} } \right)}}$$

where Cp is the curve of predicted values and Cgt is the curve of ground truth. $area\left( {C_{p} \cap C_{gt} } \right)$ represents the area of overlap and $area\left( {C_{p} \cup C_{gt} } \right)$ indicates the area of union as shown in Fig. 1. To obtain the area, an approximation of the area to the x-axis from the curve is made by dividing the space into rectangles and summing the areas of those triangles. The formula for curve area is shown as below:

2 $$area = \mathop \sum \limits_{i = 1}^{n} C(x_{i} )\Delta x$$

The accuracy of the curve area increases with the number of squares. $\Delta x$ is assigned to 0.1. We evaluated our system with different evaluation metrics such as root mean squared error (RMSE) and concordance correlation coefficient (CCC). RMSE is a standard way to measure the error of a model in predicting quantitative data. Formally, it is defined as follows:

3 $$RMSE = \sqrt {\mathop \sum \limits_{i = 1}^{n} \frac{{\left( {\widehat{{y_{i} }} - y_{i} } \right)^{2} }}{n}}$$

where $\widehat{{{\varvec{y}}_{{\varvec{i}}} }}$ is predicted value, ${\varvec{y}}_{{\varvec{i}}}$ is ground truth, and n is the number of observations. Concordance correlation coefficient (CCC) is the concordance between a predicated value and a ground truth. CCC is defined as

4 $$\rho_{C} = \frac{{2\rho \sigma_{{\hat{y}}} \sigma_{y} }}{{\rho_{{\hat{y}}}^{2} + \rho_{y}^{2} + \left( {\mu_{{\hat{y}}} - \mu_{y} } \right)^{2} }}$$

Near ± 1 is perfect concordance and 0 is no correlation.

### Evaluation

To evaluate the predictive accuracy of the survival time, the IoU was measured using (1). The results of comparisons of the performance of the proposed method with other methods were as shown in Table 2. As observed, the proposed method has better performance compared to the other models. However, the results of the Lung1 data set were not as good as those of the other datasets. The lack of correlation between input features and survival time was assumed because the CT images provided by Lung1 data set were not used.

**Table 2:** Intersection over union (IoU)

Method	DeepSurv + Exponential [1]	DeepSurv + Weibull [1]	DeepSurv + Gompertz [1]	proposed
METABRIC	0.443	0.278	0.373	0.734
Heart	0.357	0.369	0.374	0.747
PCB	0.661	0.754	0.636	0.820
Lung1	0.522	0.086	0.608	0.598

**Fig. 2:**
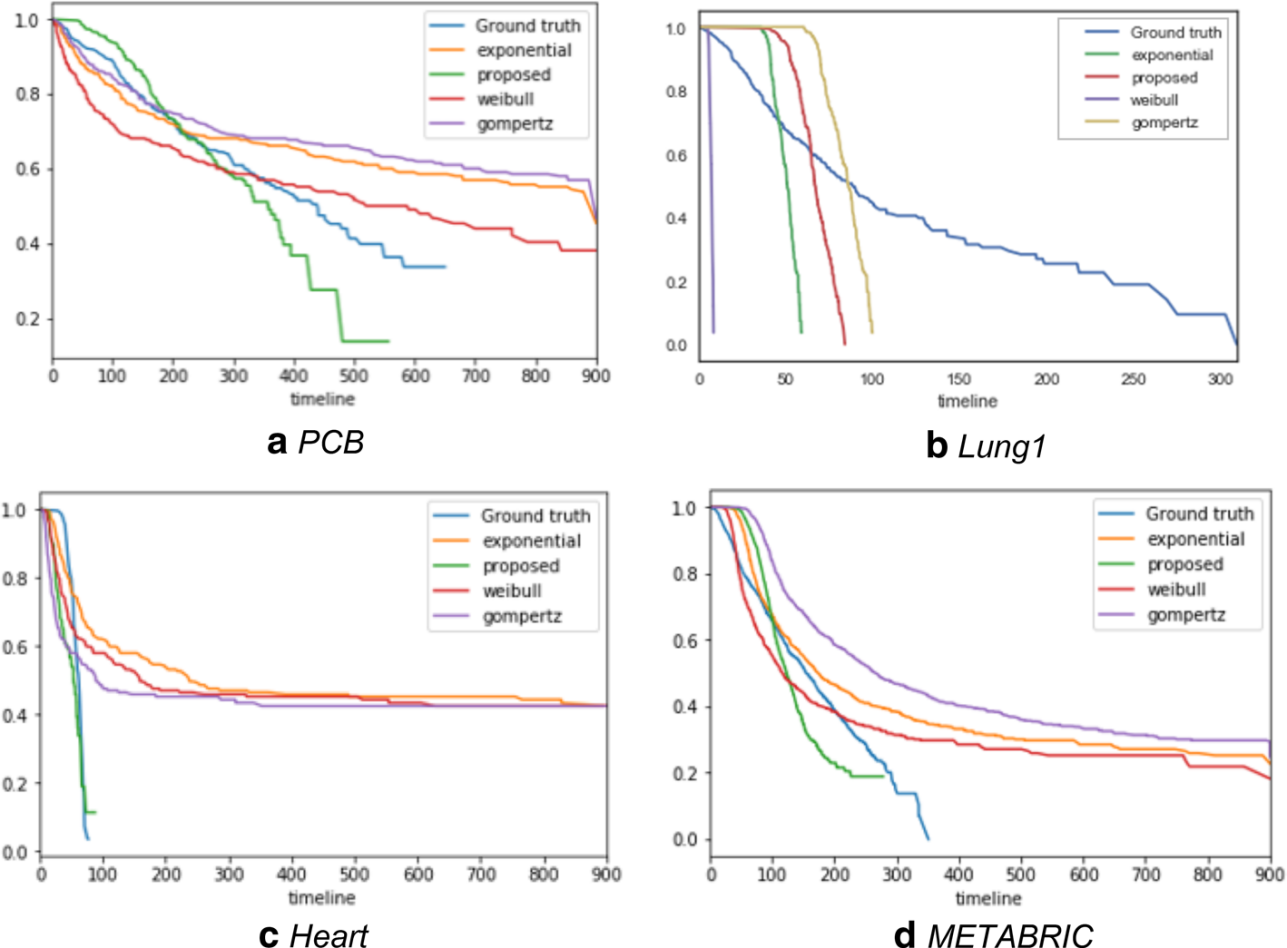
Kaplan–Meier curves for each data sets

K-M curves commonly compared two groups in researches. In other words, it is used to analyze which of any two treatments has a higher survival rate. On the other hand, we attempted to analyze a way to predict prognosis close to ground truth, rather than finding a way to have a higher survival rate. Thus, we plotted K-M curves for each algorithm to intuitively compare the performance. Figure 2 shows the Kaplan–Meier curves of methods for each data set. The curve estimated using the proposed method is closer to the ground truth curve.

**Fig. 3:**
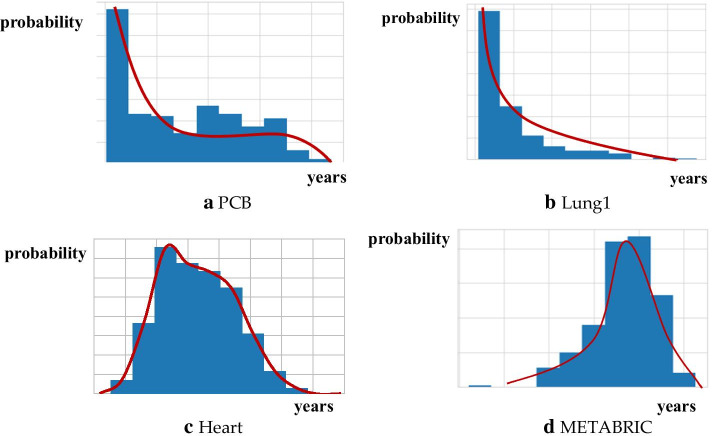
Predicted distributions for each data sets

To visualize the shape of distribution generated by the distribution function network, histograms for each data set were plotted. Figure 3 presents the histogram with the trend line. Figure 3b, d follow the exponential distribution. Figure 3a, c show the distributions close to the bell shape.

### Explainable AI for medical data

Various deep learning methods provide medical solutions by analyzing big data, however, the results are not justified. Doctors and patients cannot trust AI results unless AI give them justification. We explain our model by finding the significant factors that activates the most on the results. To find out significant factors affecting the survival ratio and help a clinician understand the relationship between risk factors and survival, we exploit 1D gradient-weighted class activation mapping (Grad-CAM) method [35]. Since conventional Grad-CAM model is limited to 2D image data, it is required to be extended to 1D to explain predictions from 1D data.

**Fig. 4:**
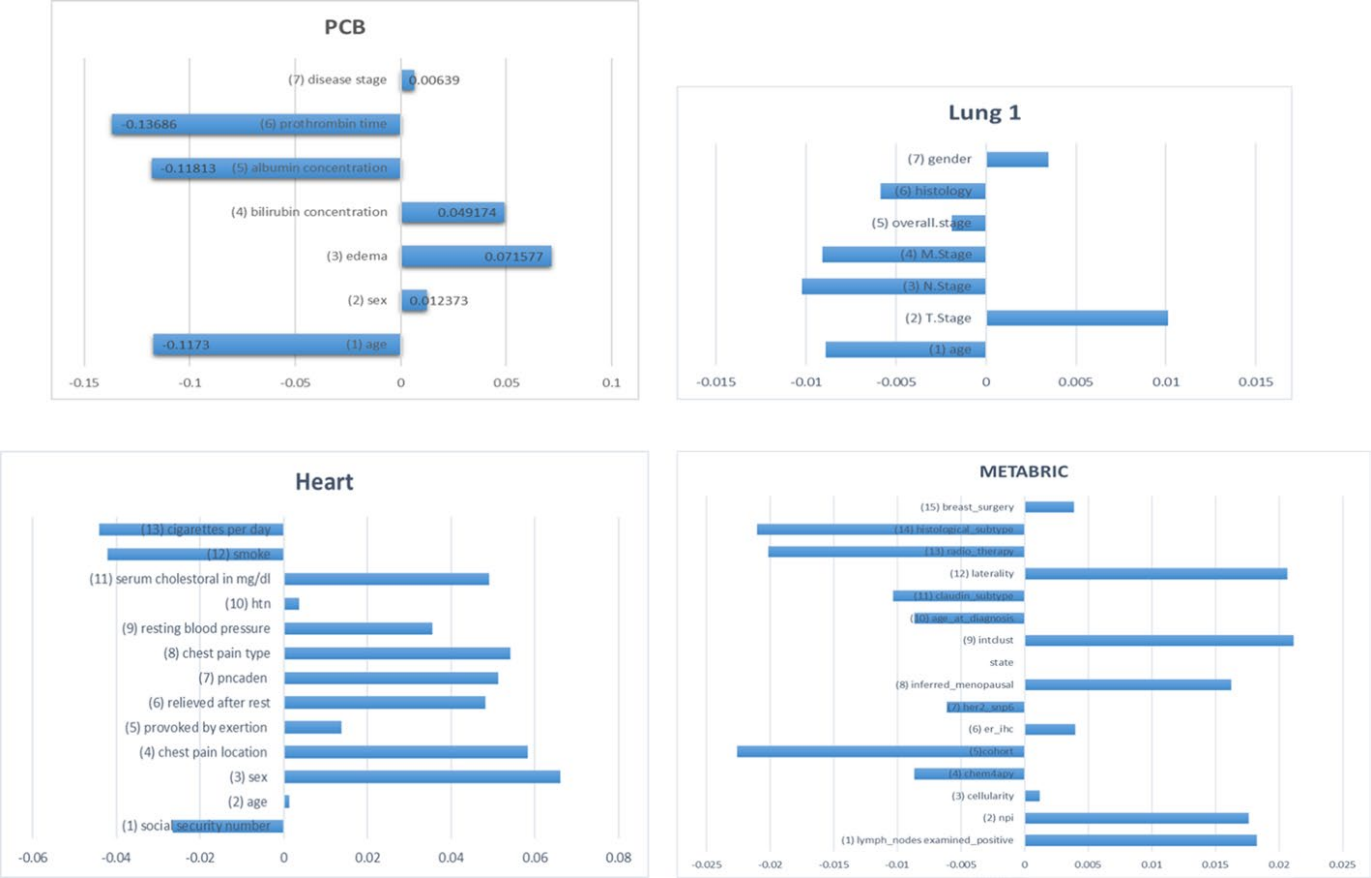
Visualization of significant features

We conducted each Grad-CAM model to obtain activation values. However, direct interpretation of the activation values is still a challenge because Grad-CAM generates the importance of each input. Thus, we averaged each activation value to analyze the significance. Figure 4 represents the averaged activation values for each dataset. According to the Fig. 4, (3) edema is the most significant factor in PCB dataset, and (7) disease stage is the least significant factor. In Lung1 dataset, (2), (3), (4) TNM stages are significant factors, but (5) overall stage is not important. In Heart dataset, (3) sex and (4) chest pain location are important features. In METABRIC dataset, (15) breast surgery is the most significant factor.

**Fig. 5:**
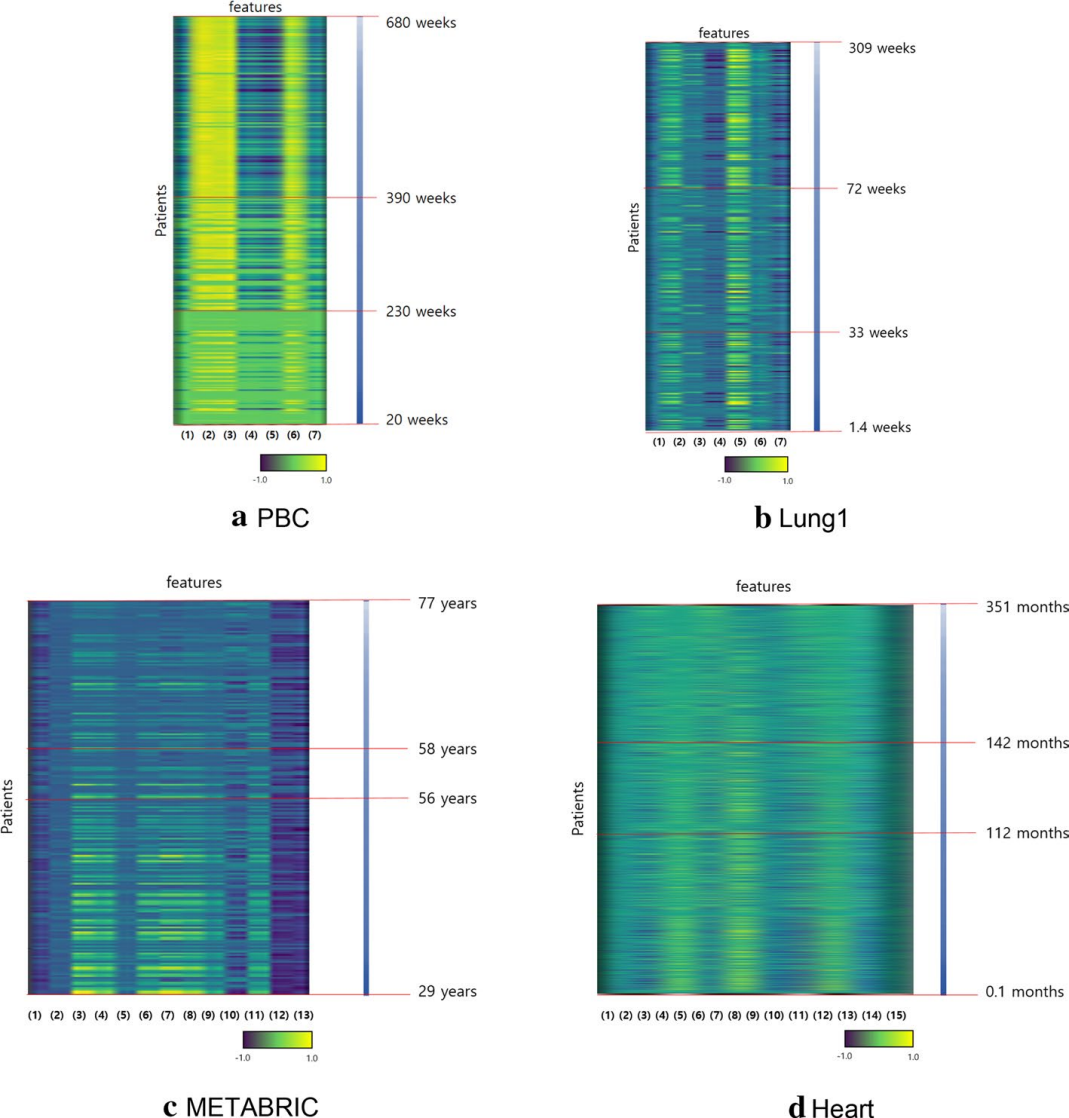
Grad-CAM heat map. Features of patients on the x axis and CAM feature expression on the y axis, revealed clusters of patients with similar expression patterns

Figure 5 shows the Grad-CAM heat map for each dataset. The heat map is sorted by survival time descending. It reveals clusters of patients separated by survival time have similar expression patterns. This can make user more reliable in the proposed method.

## Discussion

This study proposed a survival time prediction DNN architecture. Owing to censoring of the data, previous deep learning-based approaches mainly studied classification methods to determine whether patients survive rather than predict their survival time directly. As a result, this paper could be considered as the first paper to predict survival time through an end-to-end deep learning model with censoring data.

Since there were no existing methods using survival time regression deep learning, the proposed method had to be compared with the existing generation functions with the hazard rate obtained by the deep learning model [7] as shown in Table 2.

We conducted the evaluation using 2 evaluation methods: root mean squared error (RMSE) and concordance correlation coefficient (CCC). Tables 3, 4 and 5 show that our proposed method generates more accurate results compare with conventional methods.

**Table 3:** Results of root mean square error (RMSE) and concordance correlation coefficient (CCC) for PBC

Method	RMSE	CCC
CPH[5] + Exponential	227.806	0.070
CPH + Weibull	265.171	0.018
CPH + Gompertz	170.051	0.325
DeepSurv + Exponential	180.142	0.464
DeepSurv + Weibull	175.282	0.468
DeepSurv + Gompertz	218.968	0.415
Proposed	82.72	0.635

**Table 4:** Results of root mean square error (RMSE) and concordance correlation coefficient (CCC) for METABRIC

Method	RMSE	CCC
CPH + Exponential	117.838	0.206
CPH + Weibull	93.245	0.0760
CPH + Gompertz	103.713	0.228
DeepSurv + Exponential	80.753	0.172
DeepSurv + Weibull	99.722	0.228
DeepSurv + Gompertz	149.45	0.162
Proposed	56.758	0.37

**Table 5:** Results of root mean square error (RMSE) and concordance correlation coefficient (CCC) for Heart

Method	RMSE	CCC
CPH + Exponential	102.937	0.023
CPH + Weibull	46.904	0.029
CPH + Gompertz	125.893	0.0207
DeepSurv + Exponential	285.017	0.0118
DeepSurv + Weibull	158.322	0.015
DeepSurv + Gompertz	121.706	0.017
proposed	15.19	0.353

In this study, the number of features used in the experiment is small and a problem of non-convergence occurred whenever the structure of deep learning was too deep. Therefore, the structure of the proposed deep learning model does not have many layers (Table 6).

**Table 6:** Results of root mean square error (RMSE) and concordance correlation coefficient (CCC) for Lung1

Method	RMSE	CCC
CPH + Exponential	104.189	0.054
CPH + Weibull	42.019	0.037
CPH + Gompertz	50.711	0.106
DeepSurv + Exponential	293.823	0.017
DeepSurv + Weibull	298.164	0.024
DeepSurv + Gompertz	293.640	0.017
proposed	286.653	0.073

The proposed method presented an improvement in performance compared to the existing methods [15–18]. [15] utilizes the exponential curve to be a good approximation of the early survival experience. [16] uses the Weibull family, which is a special case of an exponential distribution. [17, 18] describe Hazard functions for the exponential, Weibull, gamma, Gompertz, lognormal, and log-logistic distributions. However, Lung1 dataset exhibited similar performance with an existing method. Therefore, Grad-CAM heat map cannot reveal clusters of patients with similar expression patterns as shown in Fig. 5c. On further study, it might be helpful to include imaging data to improve survival prediction.

## Conclusions

This study proposed a survival time prediction method by integrating Cox hazard network and distribution function network. First, the Cox hazard network was trained, and the distribution function network combined to generate survival time for each individual. Instead of manually selecting the distribution function to generate survival time, the proposed network trains the distribution to predict survival time. In addition, a new evaluation method to compare the area of curves was presented. The experimental results confirmed that the combination of the two networks; Cox proportional hazards network and distribution function network, can offer an effective solution for generating accurate survival time. In the future, different network architectures should be explored using multi-modality data such as CT, MRI, and clinical features to predict the survival time.

## Methods

### Survival time prediction

**Fig. 6:**
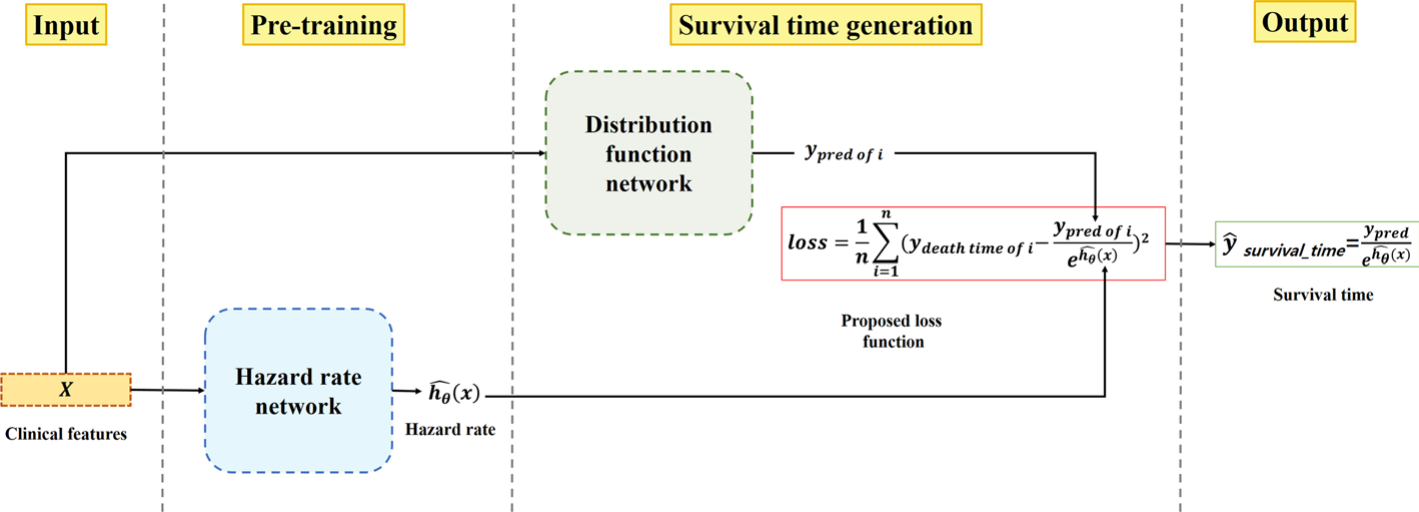
Model schematic for survival estimation

In this section, the new survival prediction method was formed by integrating hazard rate network and the proposed distribution function network. The hazard ratio network was pre-trained before merging the two networks. The pre-trained network was then integrated with the proposed distribution function network to predict death time as shown in Fig. 6.

### Hazard ratio network

To the predict hazard ratio, the architecture of DeepSurv network [1] that performs the neural network with the negative log-partial likelihood function of Cox model is applied. The Cox model is a hazard function h(t), and can be interpreted as the risk of death at time t as follows:

5 $$h\left( t \right) = h_{0} \left( t \right) \times \exp \left( {\beta_{1} x_{1} + \beta_{2} x_{2} + \cdots + \beta_{n} x_{n} } \right)$$

where, t represents the survival time, h(t) is the hazard function, (*β*_1_, *β*_2_, …,*βn*) are coefficients measuring the impact of covariates, and ho(t) is the baseline hazard function. Generally, to estimate the regression coefficients, Cox partial likelihood is optimized. The Cox partial likelihood $L\left( \beta \right)$ is given by:

6 $$L\left( \beta \right) = - \mathop \sum \limits_{{i:E_{i} = 1}} \left( {\beta x_{i} - \log \mathop \sum \limits_{{j \in R\left( {T_{i} } \right)}} e^{{\beta x_{j} }} } \right)$$

where *T*_*i*_*, E*_*i*_ are event time and event indicator for each observation, respectively and xi is a vector of clinical covariates for patient i. *R(T*_*i*_*)* is the set of patients for which no event has occurred at time t. In contrast to the conventional regression method, the hazard ratio network estimates the hazard ratio value by setting the negative log partial likelihood of (7) as loss function [1].

### Generating survival time using distribution function network

Conventional methods produce survival time using hazard ratios and specific distribution functions. Rather than generating the survival time using a particular distribution function, a method which generates the survival time through integration of a proposed distribution function network and the pre-trained hazard ratio network was proposed. To train the distribution function network, a loss function is designed to calculate the mean difference between the observation and the value obtained from the survival time generation function [6]. The proposed loss function is a variant of MSE (Mean squared error), which is the simplest and most used loss function. In addition, MSE has the advantage of being easy to understand and implement through common methods. Generally, the survival time is generated as follows:

7 $$T = \frac{u}{{e^{\beta x} }}$$

where u is the random variable with the specific mean parameter. $e^{\beta x}$ represents the hazard ratio and T is the survival time. By inserting (7) into MSE, the proposed loss function is formulated. The final loss function (Loss) is given by:

8 $$Loss = \frac{1}{n}\mathop \sum \limits_{i = 1}^{n} \left( {y_{death\,time\,of\,i} - \frac{{y_{pred\,of\,i} }}{{e^{{\widehat{{h_{\theta } }}\left( x \right)}} }}} \right)^{2}$$

where $y_{pred\,of\,i}$ represents the output of the distribution function network, y_death_ time of i is the true value of individual i, and $\widehat{{h_{\theta } }}\left( x \right)$ represents the hazard ratio from hazard ratio network.

After completing the training, the survival generation function is calculated using the predicted hazard ratio and distribution estimate. The survival generation function is defined as

9 $$\hat{y} _{survival\_time} = \frac{{y_{pred\,of\,i} }}{{e^{{\widehat{{h_{\theta } }}\left( x \right)}} }}$$

where $\hat{y} _{survival\_time}$ is the estimated survival time.

### Experimental setting

For evaluation, k-fold cross validation with k = 5 was applied [36]: the data was randomly categorized into training set (80%) and testing set (20%). Hyper-parameters were selected based on the number of features in the data set. Tables 7 and 8 presents the parameters of the hazard ratio network and the parameters of the distribution function network for each data set, respectively. Both the architecture of the two networks and the number of parameters are similar. 4-layers neural networks were used for each data set. The network was trained through Adam optimization method with a learning rate of 10–5. Xavier initialization was applied for all the layers, and a dropout probability of 0.5 was implemented only for the third layer.

**Table 7:** The parameters of the hazard ratio network

Layer	Lung1	METABRIC	Heart	PBC
Input	7	15	12	7
Layer_1	64 (Relu)	64 (Relu)	64 (Relu)	64 (Relu)
Layer_2	32 (Relu)	32 (Relu)	32 (Relu)	32 (Relu)
Layer_3	32 (Relu)	16 (Relu)	32 (Relu)	32 (Relu)
Layer_4	1	1	1	1

**Table 8:** The parameters of the distribution function network

Layer	Lung1	METABRIC	Heart	PBC
Input	7	15	12	7
Layer_1	64 (Relu)	64 (Relu)	64 (Relu)	64 (Relu)
Layer_2	32 (Relu)	32 (Relu)	32 (Relu)	32 (Relu)
Layer_3	32 (Relu)	16 (Relu)	32 (Relu)	32 (Relu)
Layer_4	1	1	1	1

To improve a model, the optimal hyper parameter values should be determined. However, it is hard to find the optimal hyper parameter. Thus, we employ a grid search method to find optimal hyper parameter. Grid search is an effective way to tune parameters in supervised learning and improve the generalization performance of a model. With grid search, we try as many combinations of the parameters of interest as possible and find the best ones. In order to find optimal parameter, we typically set the range of parameters. The combinations of the parameter are defined as

- {‘num_hidden_layers’: between 2 and 7, ‘hidden_layer_size’: between 8 and 64, ‘activation’: ['sigmoid', 'relu', 'tanh'], ‘dropout_rate’: between 0 and 0.9}

In GridSearch, we try every combination of the set of parameters defined above.

## Data Availability

Dataset name: NSCLC-Radiomics; Home page: https://wiki.cancerimagingarchive.net/display/Public/NSCLC-Radiomics; Dataset ID: None; Other requirements: None; Any restrictions to use by non-academics: None. Dataset name: Metabric breast cancer; Home page: https://ega-archive.org/datasets/EGAD00010000268; Dataset ID: EGAD00010000268; Other requirements: None; Any restrictions to use by non-academics: None. Dataset name: Heart disease; home page: https://archive.ics.uci.edu/ml/datasets/heart+disease; Dataset ID: None; Other requirements: None; Any restrictions to use by non-academics: None. Dataset name: Primary Biliary Cirrhosis (PBC) Data; Home page: https://www4.stat.ncsu.edu/~boos/var.select/pbc.html; Dataset ID: None; Other requirements: None; Any restrictions to use by non-academics: None.
